# Comparative Analysis of the One- Versus Two-Bag Method for the Treatment of Diabetic Ketoacidosis in Adults

**DOI:** 10.7759/cureus.97660

**Published:** 2025-11-24

**Authors:** Grant Morgan, Lisa Hall Zimmerman, Claudia Hanni, William B Zimmerman

**Affiliations:** 1 Department of Pharmaceutical Services, Corewell Health William Beaumont University Hospital, Royal Oak, USA; 2 Department of Urology, Michigan State University College of Osteopathic Medicine, East Lansing, USA

**Keywords:** critical care, diabetic ketoacidosis, diabetic ketoacidosis in adult, fluid management protocol, intravenous fluids, one-bag method, two-bag method

## Abstract

Introduction

Diabetic ketoacidosis (DKA) is a significant complication of diabetes mellitus, and prompt standardized management protocols are critical. In addition to insulin therapy, traditional intravenous (IV) fluid management has been provided in a one-bag method with either normal saline, lactated Ringer's, or balanced crystalloids. Several researchers have explored the use of a two-bag method of fluid administration, which includes one IV fluid bag with either normal saline, lactated Ringer's, or balanced crystalloids in addition to an IV fluid with dextrose. The findings demonstrated that patients with DKA who received the two-bag method had greater clinical benefits than patients who received the one-bag IV fluid method. This study compared the effectiveness and safety of a one-bag IV fluid administration versus a two-bag method in patients with DKA.

Methods

This retrospective study evaluated DKA patients treated with a one-bag IV fluid method at our institution compared to previously published data using one-bag and two-bag methods. Effectiveness was measured as the time to anion gap closure, pH ≥7.3, and bicarbonate ≥18 mEq/L. Safety was evaluated by occurrences of hypokalemia and hypoglycemia. Data were reported as study (S1) vs control (C1 or C2). IBM SPSS Statistics for Windows, Version 28 (Released 2021; IBM Corp., Armonk, New York, United States).

Results

Of the 598 S1 patients evaluated, 238 patients were included for analysis with a mean age of 45.4±18.9 years and Acute Physiology and Chronic Health Evaluation II (APACHE-II) of 15.1±5.8. Time to pH≥7.3 was shorter in C1 (p<0.01) and C2 (p<0.01) groups compared to S1. The mean duration of insulin infusion was shorter in both C1 (36.1±31.8 S1 vs 21.8±25.8 C1, hr; p<0.01) and C2 (36.1±31.8 S1 vs 14.1±10.7 C2, hr; p<0.01). Hospital length of stay was shorter in C1 (3.4±4.5 C1 vs 5.9±5.3 S1, days; p<0.01) and C2 (3.1±4.1 C2 vs 5.9±5.3 S1 days; p<0.01). Hypoglycemia was observed more in the S1 group (30%) compared to the C1 (10%) and C2 (3%) groups.

Conclusions

Compared to the control groups of adult patients, the S1 group was associated with increased duration of insulin infusion, risk of hypoglycemia, and prolonged hospital stay. Institution-specific DKA management protocols should be evaluated to ensure optimal treatment.

## Introduction

Diabetic ketoacidosis (DKA) is a severe complication of diabetes mellitus characterized by hyperglycemia, metabolic acidosis, and ketosis resulting from an absolute or relative insulin deficiency [1]. The treatment of DKA has a significant economic impact on the healthcare system and continues to be an evolving problem with increased healthcare costs [2,3]. A recent study estimated the incidence of admission due to DKA to be 61.7 cases per 10,000 admissions in 2017, compared to 32.0 cases per 10,000 admissions in 2003 and 53.4 cases per 10,000 admissions in 2014 [3]. Thus, strategies to decrease complications and the length of hospitalization associated with DKA are of major importance to optimize care and decrease healthcare expenditures.

Management of DKA typically requires hospital admission for treatment with intravenous insulin therapy, fluid resuscitation, and electrolyte replacement [1]. Patients with DKA are acutely volume depleted, secondary to glycosuria and osmotic diuresis [1]. Fluid therapy is essential for restoring the intravascular volume and reversing the release of counter-regulatory hormones, such as catecholamines and renin [1].

Traditionally, fluid resuscitation is initiated with isotonic crystalloids over the first hour [1]. In the one-bag fluid resuscitation method, a dextrose-containing fluid is initiated when the blood glucose is <250 mg/dL. Several limitations of this practice have been recognized, including delays in response to changing blood glucose levels, increased risk of hypoglycemia, and increased use of healthcare resources [4-6].

Studies evaluating the two-bag IV fluid method in adult patients with DKA have demonstrated a shorter time to anion gap closure and resolution of acidemia, decreased duration of insulin infusion, and lower incidence of hypoglycemia [7-9]. Given the benefits of a two-bag fluid method for the treatment of DKA in adults, we compared the effectiveness and safety of the study site's institutional one-bag fluid protocol (S1) to previously published data of the one-bag (C1) and two-bag (C2) fluid management methods.

## Materials and methods

The institutional review board at Beaumont Research Institute approved the single-center, retrospective study that evaluated adult patients admitted for DKA between 2/2019 to 12/2020 (approval no. 2021-154). Subjects were included if they were ≥18 years old, assigned an International Classification of Diseases (ICD) code for DKA, and were administered intravenous insulin infusion for at least 12 hours using the institution-approved DKA insulin infusion protocol. Subjects were excluded if the initial pH was greater than 7.3, serum bicarbonate was greater than 18 mEq/L, anion gap was less than 14 mEq/L, insulin infusion was used for an indication other than DKA, or the patient expired before discharge. Subjects were also excluded if long-acting insulin, including insulin glargine, insulin detemir, or insulin degludec, was administered less than 10 hours before the initiation of intravenous insulin infusion. Our institution utilizes a standardized insulin infusion protocol and one-bag fluid method for adult patients with DKA.

**Figure 1:**
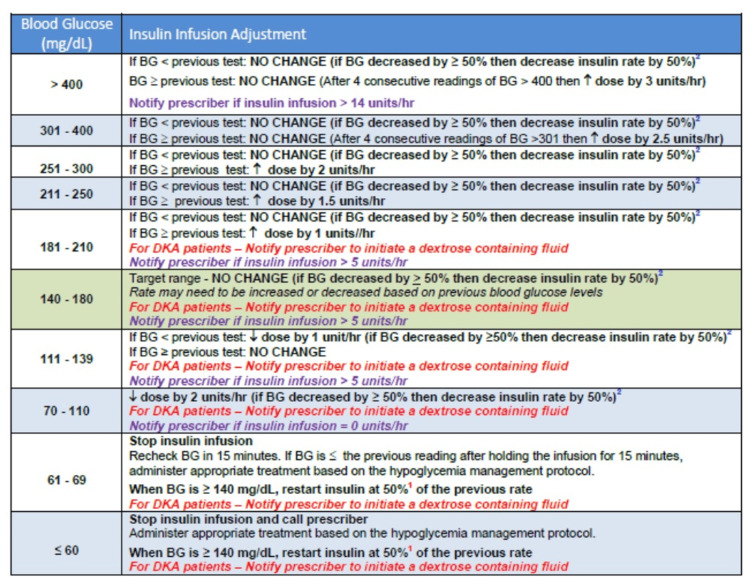
Titration of the insulin infusion based on the blood glucose measurements Source: William Beaumont University Hospital insulin protocol (reproduced with permission).

The standardized insulin infusion protocol is initiated based on the patient's serum glucose as follows: insulin one unit/hour with a blood glucose 151-175 mg/dL, insulin two units/hour with a blood glucose of 176-225 mg/dL, insulin three units/hour with a blood glucose of 226-275 mg/dL, insulin four units/hour with a blood glucose 276-325 mg/dL, insulin five units/hour with a blood glucose 326-400 mg/dL, or insulin six units/hour with a blood glucose of >400 mg/dL. The insulin titration regimen is described in Figure 1.

The choice of fluid therapy resuscitation, whether it be sodium chloride, lactated Ringer's, or balanced crystalloids, the rate of infusion, and duration of fluid therapy was at the discretion of the provider. As such, the fluid therapy was not standardized at our institution.

A prior study by Haas et al. [9] compared the use of a one-bag and two-bag fluid method in a pre-post implementation design for the treatment of adult DKA. In Haas et al., patients in the one-bag group were treated with a titratable insulin infusion and intravenous fluids at the discretion of the provider. A dextrose-containing fluid was added when the blood glucose was <250 mg/dL. In the two-bag group, patients were treated with a non-titratable insulin infusion and intravenous fluids, which consisted of two bags of 0.45% sodium chloride: one bag with 10% dextrose and the other bag with no dextrose. The standardized ratio of these bags was adjusted based on hourly blood glucose checks to maintain a total fluid rate of 250 mL/hr. This study was used as a control for our analysis. Our study group (S1) was compared to the one-bag (C1) and two-bag method (C2) groups as described in the study by Haas et al. [9]. Our study design, inclusion and exclusion criteria, and data variables were similar to Hass et al.

The primary effectiveness outcome was the time to anion gap closure. Secondary effectiveness outcomes included time to pH normalization, time to serum bicarbonate normalization, duration of insulin infusion, and length of hospital stay. Safety outcomes included the incidence of hypoglycemia (<70 mg/dL) and incidence of hypokalemia (<3.3 mEq/L) during the first 96 hours of admission. Additional endpoints evaluated included the total intravenous fluid volumes administered at 24 hours, 48 hours, 72 hours, and 96 hours from admission for the study group. Volumes were calculated using the documentation of fluid boluses and maintenance fluid rates in the electronic health records. The transition to dextrose-containing fluid was evaluated as the period between the time of order entry of the dextrose-containing fluid and the time of administration on the electronic health record. Baseline characteristics, past medical history, and laboratory values at the time of admission were used to calculate the Charlson Comorbidity Index and Acute Physiology and Chronic Health Evaluation II (APACHE II) scores [10,11]. 

The severity of DKA was based on the initial arterial pH or a venous pH if the arterial pH was not available. DKA severity was adapted from the American Diabetes Association consensus statement on hyperglycemic crises in adult patients with diabetes [1]. Subjects with an initial arterial blood gas revealing a pH of 7.25 to 7.29 were classified as mild, a pH of 7.0 to 7.24 as moderate, and a pH of <7.0 as severe. Closure of the anion gap was defined as a calculated anion gap ≤12 mEq/L. Normalization of pH was defined as an arterial pH ≥7.3, and normalization of bicarbonate was defined as a serum bicarbonate ≥18 mEq/L. Hypoglycemia was defined as an episode of either a point-of-care or a serum glucose <70 mg/dL. Hypokalemia was defined as an episode of serum potassium <3.3 mEq/L.

Using an estimated difference of 25% between groups, a sample size of 199 subjects was calculated to achieve 80% power [7-9]. The McNemar test was used for analysis of categorical and nominal data. Mann-Whitney U was used for the analysis of continuous data. Estimated means and standard deviations were utilized from the historical control by Haas et al. for statistical analysis. A subgroup analysis of the study group (S1) patients who developed hypoglycemia (<70 mg/dL) versus those who did not develop hypoglycemia was also done. IBM SPSS Statistics for Windows, Version 28 (Released 2021; IBM Corp., Armonk, New York, United States) was used for analysis with a p-value of <0.05 being considered significant.

## Results

**Figure 2:**
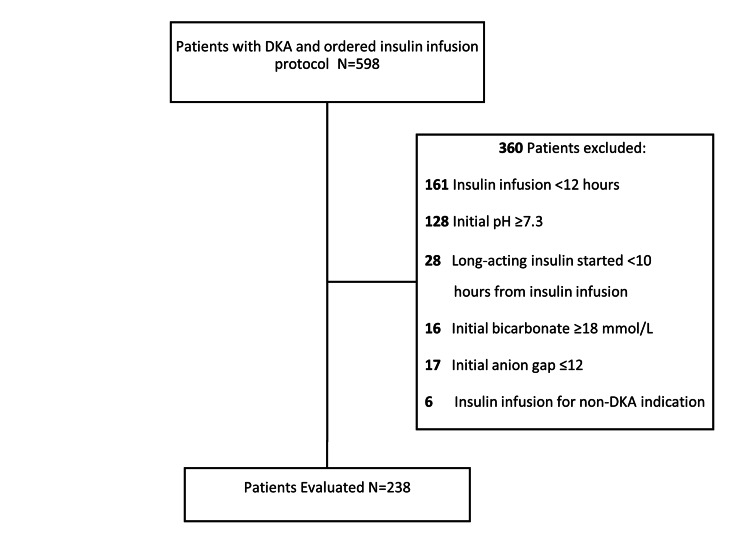
The consort diagram of patients included in the study group

After identifying 598 patients with DKA in the study group, 238 met the eligibility criteria and were included in the study cohort (Figure 2).

The C1 and C2 groups consisted of 107 and 68 patients, respectively. Overall, baseline characteristics (Table 1) were similar between the study group (S1) and the one-bag (C1) and two-bag (C2) control groups.

**Table 1:** Baseline demographics of the study population ^*^Standard deviations calculated from reported 95% confidence interval from the historical control group, Haas et al. [9]; nominal data analyzed with a McNemar test; continuous variables evaluated with Mann-Whitney U test, U-value unavailable with S1 being compared to historical controls, C1 and C2 [9] APACHE II: Acute Physiology and Chronic Health Evaluation; DKA: Diabetic ketoacidosis; ICU: Intensive care unit; SD: Standard deviation.

Characteristic	Study group (S1); N=238	One-bag control group (C1)^*^; N=107	Two-bag control group (C2)^*^; N=68
Age (years), mean ± SD^ⴕ^	45.4 ± 18.9	34.8 ± 17.2	32.4 ± 13.8
Male, n (%)	105 (44)	49 (45)	28 (41)
Weight (kg), mean ± SD	79.5 ± 25.2	-	-
Body mass index (kg/m^2^), mean ± SD	27.3 ± 7.4	-	-
Charlson Comorbidity Index, mean ± SD	1.5 ± 1.0	-	-
APACHE-II Score, mean ± SD	15.1 ± 5.8	-	-
ICU Admission, n (%)	126 (53)	-	-
Medical ICU, n (%)	77 (61)	-	-
Surgical ICU, n (%)	26 (21)	-	-
Cardiac ICU, n (%)	23 (18)	-	-
Initial pH, mean ± SD	7.14 ± 0.1	7.11 ± 0.1	7.12 ± 0.1
Initial bicarbonate (mmol/L), mean ± SD	8.7 ± 3.6	9.6 ± 3.9	10.3 ± 6.6
Initial anion gap (mEq/L), mean ± SD	26.7 ± 6.2	27.6 ± 6.3	26.9 ± 6.6
Initial serum glucose (mg/dL), mean ± SD^ⴕ^	633 ± 293	546 ± 230	539 ± 254
β-hydroxybutyrate (mmol/L), mean ± SD	8.4 ± 3.7	-	-
Hemoglobin A1C (mmol/L), mean ± SD	12.1 ± 2.4	-	-
DKA Severity			
Mild, n (%)	50 (21)	-	-
Moderate, n (%)	155 (65)	-	-
Severe, n (%)	33 (14)	-	-

Initial arterial pH, serum bicarbonate, and anion gap were similar across all groups. The mean age in the S1 group was 45.4±18.9 years versus 34.8±17.2 (CI -14.6 to -6.5, NS) and 32.4 ± 13.8 (-17.8 to -8.1, NS) years in the C1 and C2 groups, respectively. Nearly 79% of patients in the S1 group had moderate to severe DKA and over 53% of these patients required care in the intensive care unit. The mean Charlson Comorbidity Index and APACHE-II scores at admission of the S1 group were 1.5±1.0 and 15.1±5.8, respectively.

Overall, the time to anion gap closure in the S1 group was 18.5±15.2 hours (Table 2).

**Table 2:** Summary of the primary and secondary clinical outcomes ^*^Standard deviations calculated from reported 95% confidence interval from the historical control group, Haas et al. [9]; nominal data analyzed with a McNemar test; continuous variables evaluated with the Mann-Whitney U test, U-value unavailable with S1 being compared to historical controls, C1 and C2 [9]; p-value <0.05 considered statistically significant. SD: Standard deviation.

Characteristic	Study group (S1); N=238	One-bag control group (C1)^*^; N=107	P-value	Two-bag control group (C2)^*^; N=68	P-value
Time to anion gap closure (hours), mean ± SD	18.5 ± 15.2	-	-	-	-
Time to pH≥7.3 (hours), mean ± SD	14.8 ± 9.5	10.8 ± 5.7	<0.01	12.4 ± 4.8	0.04
Time to bicarbonate ≥18 mEq/L (hours), mean ± SD	22.7 ± 14.1	20.0 ± 20.6	0.16	13.4 ± 8.9	<0.01
Duration of insulin infusion (hours), mean ± SD	28.1 ± 11.7	21.8 ± 25.8	<0.01	14.1 ± 10.7	<0.01
Length of stay (days), mean ± SD	5.9 ± 5.3	3.4 ± 4.5	<0.01	3.1 ± 4.1	<0.01
Time to initiation of long-acting insulin (hours), mean ± SD	27.8 ± 12.7	21.6 ± 23.0	<0.01	14.7 ± 4.8	<0.01
Time to initiation of dextrose-containing fluid (hours), mean ± SD	13.3 ± 10.6	-	-	-	-
Hypoglycemia, n (%)	71 (30)	11 (10)	<0.01	2 (3)	<0.01
Hypokalemia, n (%)	69 (29)	29 (27)	0.72	11 (16)	<0.01

The S1 group had a slower resolution of acidemia compared to the C2 group (14.8±9.5 S1 vs 12.4±4.8 C2, hours; p=0.04). This was consistent with the time to serum bicarbonate ≥18 mEq/L (22.7±14.1 S1 vs 13.4±8.9 C2, hours; p<0.01). The duration of insulin infusion was longer in the S1 group (28.1±11.7 S1 vs 14.1±10.7 C2, hours; p<0.01), and the S1 group had a longer mean hospital length of stay (5.9±5.3 S1 vs 3.1±4.1 C2, days; p<0.01) compared to the C2 group. With respect to safety (Table 2), an increased incidence of hypoglycemia was more likely to occur in the S1 group compared to the C2 (30% S1 vs 3% C2; p<0.01), and the S1 group had an increased incidence of hypokalemia (29% S1 vs 16% C2; p=0.03).

Comparing the S1 and the C1 groups, the S1 group had a slower resolution of correction of the acidemia (14.8±9.5 S1 vs 10.8±5.7 C1, hours; p<0.01), a longer duration of insulin infusion (28.1±11.7 S1 vs 21.8±25.8 C1, hours; p<0.01), a longer hospital length of stay (5.9±5.3 S1 vs 3.4±4.5 C1, days; p<0.01), and an increased incidence of hypoglycemia (30% S1 vs 10% C1; p<0.01). No difference was observed in the time to serum bicarbonate ≥18 mEq/L (p=0.16) or the incidence of hypokalemia (p=0.72) in the S1 group compared to C1.

**Figure 3:**
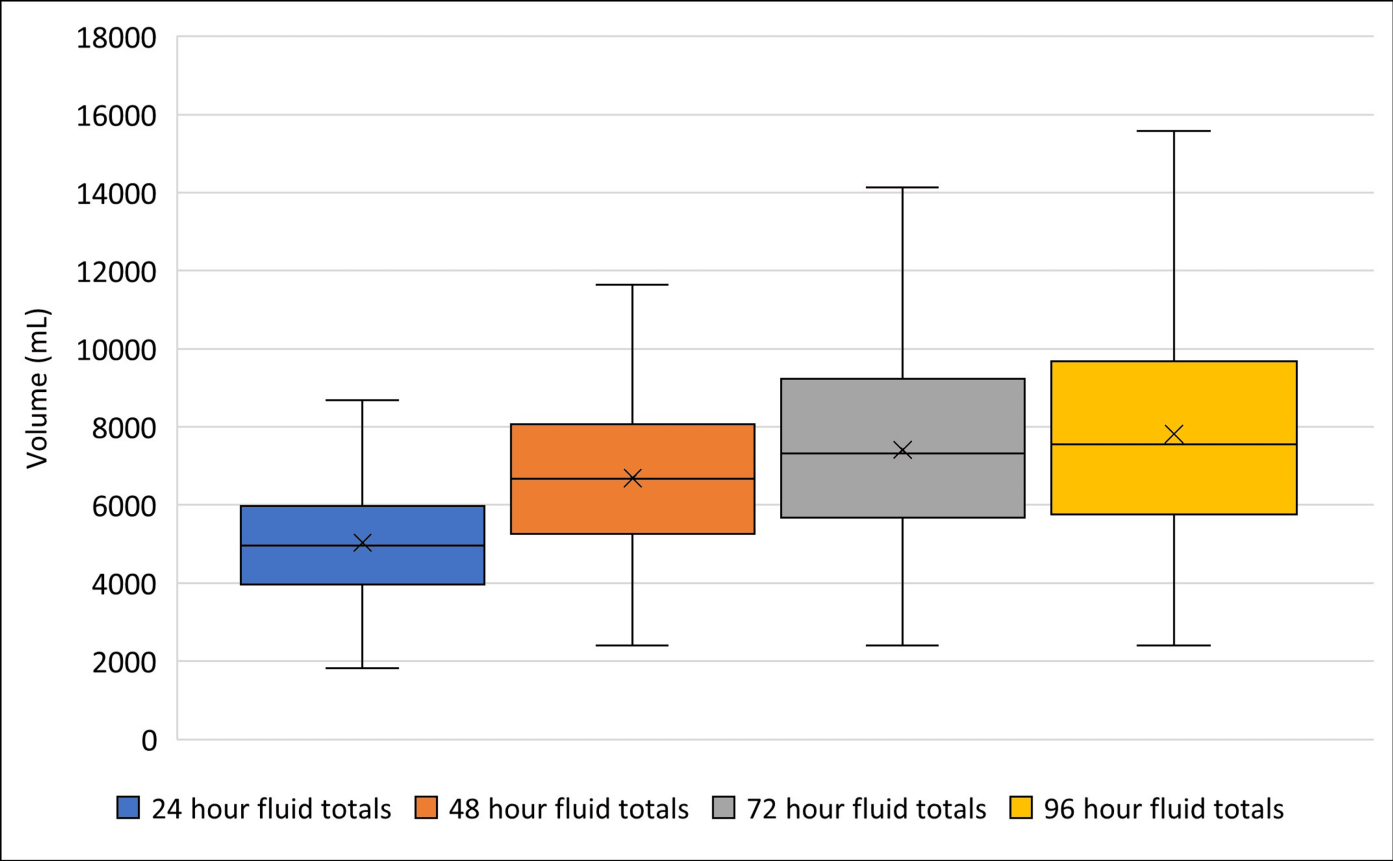
The total volume of intravenous fluids within the first 24, 48, 72, and 96 hours after presentation (S1 group)

The median volume of intravenous fluid administered (Figure 3) in the S1 group in the first 24 hours from presentation was approximately 4.8 L (IQR 4.0, 6.0).

The median fluid volume at 48, 72, and 96 hours from presentation was 6.5 L (IQR 5.3, 8.1), 7.1 L (IQR 5.3, 9.2), and 7.4 L (IQR 5.8, 9.7), respectively. The time to first dextrose containing fluid in the S1 group was 13.3±10.6 hours.

In the subgroup analysis of the S1 group (Table 3), 71 patients (30%) developed hypoglycemia versus 167 patients (70%) without hypoglycemia.

**Table 3:** Description of the study group (S1) patients with diabetic ketoacidosis (DKA) who developed hypoglycemia compared to those without hypoglycemia Nominal data analyzed with a McNemar test; continuous variables evaluated with the Mann-Whitney U test; a p-value <0.05 considered statistically significant. ICU: Intensive care unit; APACHE-II: Acute Physiology and Chronic Health Evaluation II.

Characteristic	Hypoglycemia; N=71	No hypoglycemia; N=167	P-value
Age (years), mean ± SD	46.3 ± 20.9	44.3 ± 18.0	0.46
Female, n (%)	48 (68)	85 (51)	0.02
Male, n (%)	23 (32)	82 (49)	0.02
Weight (kg), mean ± SD	67.1 ± 16.3	84.8 ± 25.0	<0.01
Body mass index (kg/m^2^), mean ± SD	23.5 ± 4.9	29.0 ± 8.2	<0.01
Charlson Comorbidity Index, mean ± SD	1.8 ± 1.6	1.3 ± 1.3	0.03
APACHE-II Score, mean ± SD	15.8 ± 5.8	14.7 ± 5.8	0.18
ICU admission, n (%)	40 (56)	86 (52)	0.49
Initial pH, mean ± SD	7.13 ± 0.14	7.15 ± 0.11	0.32
Initial bicarbonate (mmol/L), mean ± SD	8.7 ± 3.5	8.7 ± 3.6	0.97
Initial anion gap (mEq/L), mean ± SD	27.7 ± 7.0	26.3 ± 5.8	0.10
Initial serum glucose (mg/dL), mean ± SD	694 ± 309	607 ± 283	0.04
β-hydroxybutyrate (mmol/L), mean ± SD	8.4 ± 4.2	8.4 ± 3.5	0.95
Hemoglobin A1C (mmol/L), mean ± SD	11.3 ± 2.4	12.5 ± 2.3	<0.01
DKA severity			
Mild, n (%)	15 (21)	35 (21)	0.98
Moderate, n (%)	41 (58)	114 (68)	0.12
Severe, n (%)	15 (21)	18 (11)	0.03

**Figure 4:**
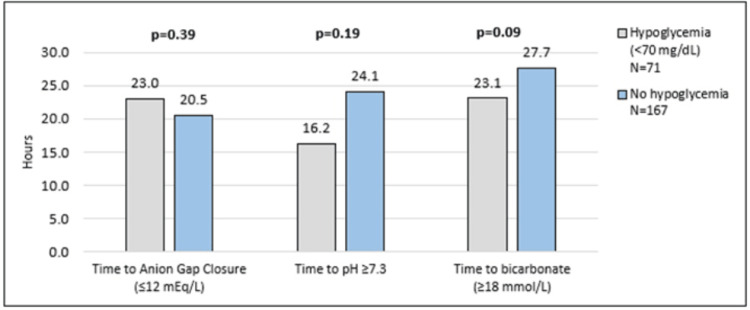
A description of time to the associated primary and secondary outcomes in the hypoglycemia versus no hypoglycemia groups in the study group (S1) patients A p-value <0.05 was considered significant.

Patients who developed hypoglycemia were more likely to be female (68% hypoglycemia vs 51% no hypoglycemia, p=0.02), to have a lower body mass index (23.5 hypoglycemia vs 29.0 no hypoglycemia, kg/m^2^; p<0.01), and present with severe DKA (21% hypoglycemia vs 11% no hypoglycemia; p=0.03). No differences were found in the time to anion gap closure, the time to pH normalization, or the time to bicarbonate normalization between groups (Figure 4).

Of the patients who developed hypoglycemia, 52% of episodes occurred while receiving an intravenous insulin infusion. Hypoglycemia between the transition to dextrose-containing fluid was observed in only 5% of patients in the S1 group.

## Discussion

DKA continues to be a significant cause of hospitalization in the United States, with nearly 200,000 admissions annually [2,3]. Over the past decade, the rates of in-hospital mortality from DKA have decreased, with rates at 0.33% in 2014 [3]. This decrease has been at least partially attributed to the recognition of prompt, standardized treatment protocols for such patients [2,3]. However, recent studies have observed an increase in mortality rates, which have now been estimated to be 0.38%. Updates to institutional DKA guidelines and order sets are important to ensure safe and effective treatment in this patient population [3].

Acute treatment of DKA requires frequent monitoring of serum glucose, electrolytes, and volume status, which often constitutes changes in the fluid administered, concentration of fluid additives, and fluid administration rates. In the traditional one-bag method of fluid administration, the initiation of dextrose-containing fluids can be delayed through the logistics of ordering in the electronic health record, preparation and delivery of the fluid, and initiation of the dextrose-containing fluid to the patient while insulin is continuously infusing. The two-bag method of fluid administration offers significant advantages, allowing for rapid, standardized alterations in fluid therapy, especially as it relates to the initiation of dextrose-containing fluid based on the serum glucose assessment.

Although most of the available data of the two-bag method are in the pediatric population [4], an increasing number of studies in the adult population have shown that the this method can decrease the delay in the transition of fluids, can decrease in the total volume of fluids administered, and can allow for safer continuation of uninterrupted insulin therapy [5-9]. In addition, the decreased risk of hypoglycemic events in the two-bag method holds significant value, given that in patients treated with insulin, hypoglycemia is associated with an increased risk of inpatient mortality and increased length of hospital stay [12]. The results of our study replicated many of the results shown in previous studies, including increased effectiveness and safety in the treatment of DKA when using the two-bag method.

Nahle et al. retrospectively evaluated adult patients with DKA in a two-bag method and found an improvement in the time to DKA resolution [13]. Haas et al. also followed up with a larger cohort of patients and demonstrated a short time to DKA resolution [14]. Halfon et al. evaluated a nurse-driven adult DKA treatment protocol in a one-bag versus two-bag fluid administration method, which resulted in an improved time to DKA resolution with the latter [15]. Furthermore, Gilchrist et al. compared before versus after implementation of the two-bag fluid order set for DKA management, which resulted in an improved time to DKA resolution [16]. Our study also demonstrated that the historical control two-bag (C2) method had a shorter time to DKA resolution than the one-bag (S1) group. In addition, Munir et al. [7], in a retrospective study of one-bag versus two-bag method for the treatment of adult DKA, observed significant improvement in the time to anion gap closure and improved time to euglycemia in the two-bag fluid group. Gilchrist et al. also found a shorter time to anion gap closure in the two-bag method [16]. Our study demonstrated a longer time to anion gap closure in the one-bag method in the study group (S1) versus the historical control of a two-bag method (C2).

While reviewing time to bicarbonate normalization, Haas et al. [9] showed that the two-bag fluid administration method had a significant advantage over the one-bag method, and resulted in a shorter time to bicarbonate normalization. In Haas et al.'s follow up study, the authors found a short time to bicarbonate normalization in the two-bag group [14]. Comparatively, our study revealed a longer time to bicarbonate normalization with the one-bag study group (S1) compared to the historical controls (C1 and C2).

Evaluating the duration of continuous insulin administration in the early management of DKA, both trials from Haas et al. [9,14] showed that the two-bag method had significant advantages over the one-bag method, which included a shorter duration of insulin infusion. Nahle et al. [13] and Gilchrist et al. [16] also demonstrated a shorter duration of insulin drip therapy with the two-bag method. Our study showed similar findings.

Any occurrence of hypoglycemia can lead to catastrophic effects, and it can occur in the acute management of DKA with continuous infusion of insulin therapy. Both Munir et al. [7] and Haas et al. [9] found a significant decrease in hypoglycemic events in the two-bag group. In the follow-up study by Haas et al. (2023), involving a larger number of patients, hypoglycemia was reduced with the two-bag method [14]. Other researchers, Halfon et al. [15], Moorhouse et al. [17], and Gilchrist et al. [16], also reported a decrease in hypoglycemic events with the two-bag fluid method. In the present study, the two-bag historical control had a lower incidence of hypoglycemia compared to the one-bag study group. Similarly, Haas et al. [9] showed that a two-bag method had significant advantages over a one-bag method, which included decreased rates of hypokalemia. This finding has been replicated in studies by Nahle et al. [13], Haas et al. [14], Halfon et al. [15], Moorhouse et al. [17], and Adams et al. [18]. Our study also demonstrated a decrease in hypokalemic events in the two-bag historical control versus the one-bag study group (S1).

Our study offered a real-world experience in a large cohort of adult patients with DKA that utilized a standardized insulin infusion and traditional one-bag method of fluid administration for the treatment of DKA. This was compared to two historical comparison groups. In the present study, the one-bag group (S1) demonstrated a slower resolution of acidemia, a prolonged duration of insulin infusion, and an increased incidence of hypoglycemia, likely secondary to variability in prescriber practice, apart from the lack of the use of the two-bag method. These findings necessitated the reevaluation of the DKA treatment guidelines at our institution, especially with regards to fluid administration and insulin therapy.

Several limitations of this study should be considered. The design was developed using similar inclusion and exclusion criteria as described from Haas et al. [9] with the intention of decreasing potential bias, however differences in the patient populations, each site's insulin infusion protocols, and local practices may have influenced the differences seen in our analysis. Another limitation is that we did not have actual patient data from Haas et al. [9] and conducted our statistical analysis using the reported mean and 95% confidence intervals for comparisons. In addition, the frequency and timing of laboratory collection may have also influenced the outcomes measured in this study as these variables were at the discretion of the provider. The choice of laboratory assessment was likely also different between the study site and the historical control study and was also not controlled. The results of the current study’s effectiveness outcomes may be altered, considering time zero was not reported in the historical control groups, whereas our study used the time of admission as time zero. 

We aimed to have the S1 group represent a traditional DKA management strategy when long-acting subcutaneous insulin is initiated at the resolution of DKA. Hence, we excluded patients who received subcutaneous insulin less than 10 hours from the start of intravenous insulin infusion. The staging of DKA severity is multifactorial with considerations for laboratory data and subjective evaluation of mental status which was difficult to ascertain in a retrospective fashion. Lastly, the choice of initial and subsequent fluid type and administration rates were not controlled and were based on the provider's discretion due to patient-specific factors. Future studies should attempt to control many of these variables to properly evaluate the true clinical benefits of the two-bag fluid administration method.

## Conclusions

DKA continues to be a significant problem in patients with diabetes mellitus. Insulin therapy, intravenous fluids, and electrolyte management remain the foundation of acute DKA treatment. DKA treatment guidelines have the potential to reduce further complications. Our institution currently utilizes a standardized insulin infusion, a one-bag fluid administration method, and electrolyte replacements for the clinical management of patients with DKA. Reported studies demonstrate that an alternative two-bag fluid management strategy has clinical and logistical benefits for the treatment of DKA. In this study, the effectiveness and safety of the two-bag method improved outcomes compared to the one-bag method. Continuous evaluation and optimization of institution-specific DKA management protocols should be performed to ensure the most effective and safe treatment of DKA.
